# Higher prevalence of dupilumab‐induced ocular adverse events in atopic dermatitis compared to asthma: A daily practice analysis

**DOI:** 10.1002/clt2.12386

**Published:** 2024-08-16

**Authors:** Anne R. Schlösser, Lotte Bult, John C. Thelen, Alberta A. H. J. Thiadens, Renske Schappin, Tamar E. C. Nijsten, Johannes C. C. M. in 't Veen, Gerrit J. Braunstahl, DirkJan Hijnen

**Affiliations:** ^1^ Department of Dermatology Erasmus MC University Medical Center Rotterdam The Netherlands; ^2^ Department of Pulmonology Franciscus Gasthuis and Vlietland Rotterdam The Netherlands; ^3^ Department of Pulmonology Erasmus MC University Medical Center Rotterdam The Netherlands; ^4^ Department of Ophthalmology Erasmus MC University Medical Center Rotterdam The Netherlands

**Keywords:** atopic dermatitis, dupilumab, dupilumab ocular surface disease, severe asthma, T2‐inflammation

## Abstract

**Background:**

Dupilumab has been shown to be an effective treatment in moderate‐to‐severe atopic dermatitis (AD) and severe asthma (SA). However, comparative real‐world analyses of adverse events (AE), particularly dupilumab‐associated ocular surface disease (DAOSD), are lacking.

**Objective:**

This is the first real‐world study to provide insight into the prevalence of AEs associated with dupilumab in AD compared with SA. Secondary objectives were to assess the prevalence, onset and therapeutic strategies of DAOSD and evaluate dupilumab discontinuation rates.

**Methods:**

Data from two daily practice registries including AD and SA patients receiving dupilumab treatment were analyzed. Adverse events, including DAOSD, were evaluated.

**Results:**

In total, 322 AD and 148 SA patients were included. Headaches (23.6%), injection site reactions (10.1%), and influenza‐like symptoms (13.5%) were more prevalent in SA patients. Interestingly, ocular AEs were significantly more prevalent in AD patients (62.1%, *p* < 0.001), including conjunctivitis (17.1%, *p* = 0.004). 88% AD and 47% SA patients with ocular AEs received one or more ophthalmic treatment(s). Additionally, 20% of AD and 17.6% of SA patients discontinued dupilumab treatment due to ocular AEs, while only 65% of these AD and none of these SA patients were referred to an ophthalmologist.

**Conclusion:**

The higher incidence of DAOSD in AD patients compared with SA patients in this real‐world study highlights the importance of physician awareness, especially when prescribing dupilumab to AD patients. Conversely, the findings of this study help alleviate potential concerns about ocular AEs in patients with SA who do not have comorbid AD. Furthermore, the effective management of most ocular AEs with ophthalmic treatments suggests favorable tolerability of dupilumab in daily practice, and multidisciplinary collaboration is essential to proactively manage ocular AEs before discontinuing dupilumab.

## 1 INTRODUCTION

Atopic dermatitis (AD) and asthma are chronic inflammatory diseases that often coexist and have a large impact on the quality of life of patients. Both AD and asthma are often characterized by type 2 (T2) dominated inflammation. Dupilumab was the first biological agent that was registered for the treatment of both AD and severe asthma (SA).^1^, ^2^ Dupilumab is a fully human monoclonal antibody that blocks the interleukin‐4 receptor alpha chain, inhibiting the binding of IL‐4 and IL‐13, thereby effectively inhibiting T2 inflammation.^3^, ^4^ Clinical trials and real‐world studies have shown that dupilumab improves signs and symptoms as well as the quality of life of AD patients. In patients with SA, dupilumab has been shown to improve asthma control and lung function, reduce the number of exacerbations, and decrease the use of oral corticosteroids (OCS).^4^, ^5^, ^6^, ^7^, ^8^, ^9^

Although the number of adverse events (AE) in clinical trials with dupilumab in AD was relatively low in daily practice, the incidence of dupilumab‐associated AEs, and particularly dupilumab‐associated ocular surface disease (DAOSD) was more frequently reported with incidences up to 34.2%. DAOSD includes manifestations such as conjunctivitis and blepharoconjunctivitis.^10^, ^11^, ^12^ Interestingly, in clinical trials with dupilumab for other T2‐driven chronic inflammatory diseases, such as asthma, increased incidence of DAOSD was not reported.^10^ In daily practice, the incidence of DAOSD in SA patients was up to 10.8%.^9^ The higher prevalence of DAOSD in AD patients suggests that patients with AD, but not SA, are predisposed to develop DAOSD.

Not all published safety data on dupilumab may fully reflect daily clinical practice, mainly due to strict inclusion and exclusion criteria of clinical trials.^13^ The aim of this study was to investigate AEs of dupilumab treatment in real‐life in AD patients compared with SA patients, with a special focus on ocular AEs. Additionally, the prevalence, onset and management of ocular AEs and discontinuation of dupilumab treatment due to ocular AEs were evaluated.

## 2 METHODS

### 2.1 Study design and population

This study included patients from two databases: one with patients diagnosed with AD and one with patients diagnosed with SA. All adult patients diagnosed with moderate to severe AD who started dupilumab treatment at the Erasmus MC University Medical Center (EMC) (Rotterdam, The Netherlands) between October 2017 and November 2022, enrolled in the Immune‐Mediated Inflammatory Disorder (IMID) registry, a prospective AD cohort, were included in this study. In addition, all adult patients diagnosed with SA who started dupilumab treatment at the Franciscus Gasthuis & Vlietland (FGV) Hospital (Rotterdam, The Netherlands) between January 2019 and December 2020, enrolled in the RelyOnDupi registry, a retrospective SA cohort, were included in this study. The IMID registry and RelyOnDupi registry were both approved by the local medical ethics committee (EMC: MEC‐2017‐1123 and FGV: FGV‐2021‐042 respectively), and all patients provided informed consent.

To be eligible for inclusion in this study, patients had to be diagnosed with moderate to severe AD^14^ or uncontrolled SA,^2^ and had initiated dupilumab treatment. Patients had to be at least 18 years old at the start of dupilumab treatment. Exclusion criteria were lack of informed consent, dupilumab treatment for a T2‐related condition other than AD or SA (nasal polyposis), or discontinuation of dupilumab treatment within 4 weeks.

### 2.2 Treatment and data collection

AD patients were treated with dupilumab 300 mg every 2 weeks after a loading dose of 600 mg. Patients visited the outpatient clinic at 4 weeks, 12–16 weeks, and every three months after the start of dupilumab treatment. SA patients were treated with 200 mg dupilumab every 2 weeks after a loading dose of 400 mg. Patients with additional T2‐comorbidities (AD or nasal polyposis) or those who were dependent on maintenance OCS therapy were treated with 300 mg every 2 weeks after a loading dose of 600 mg. During dupilumab treatment, AEs were evaluated at every visit. To classify the severity of concurrent AD in SA patients and asthma in AD patients, we developed a medication‐based grouping approach. For AD severity categorization, we designated patients as having “Mild AD” if they only used class I/II topical corticosteroids (TCS). Patients using TCS III/IV were classified as having “”Moderate AD’, while patients requiring systemic treatment were categorized as having “severe AD.”^15^ For the classification of asthma severity in AD patients, we used the Global Initative for Asthma (GINA) guidelines: GINA step 1–2 is mild asthma, GINA step 3–4 is moderate asthma and GINA step 5 is SA.^2^

### 2.3 Outcome measures

Primary endpoints were the proportion of dupilumab‐associated AEs in AD patients compared with SA patients. Secondary objectives were to assess the prevalence, onset and treatment of ocular AEs and to investigate discontinuation of dupilumab treatment.

In the two databases, patient characteristics and atopic co‐morbidities were registered at baseline. AEs and treatment of AEs, including ocular AEs and DAOSD diagnosis were documented during follow up. Ocular AEs were categorized based on specific ophthalmic signs, including redness, irritation, tearing, itching, photophobia, swelling, dryness and blurred vision, and DAOSD diagnoses, including conjunctivitis, blepharitis and keratitis. If necessary, ophthalmological treatments, including lubricants, antihistamine, non‐steroidal anti‐inflammatory drugs (NSAID) and steroidal‐eye drops, peri‐ocular tacrolimus skin ointment and antibiotic eye ointment (chloramphenicol) were initiated. Referrals to the ophthalmologist were documented. Reasons for discontinuation of dupilumab treatment were registered, such as AEs and inadequate disease control. Eosinophil counts and weight measurements were only registered in SA patients, whereas the date of onset of ocular AEs was only documented in AD patients.

### 2.4 Statistical analysis

Numerical data were described as mean (± standard deviation (SD)) or median [interquartile range] (IQR) depending on their distribution. Categorical data were evaluated as the number and percentage of patients (*n*, %). Differences in baseline characteristics between both groups were calculated using the independent‐ or paired *T*‐test or Mann‐Whitney *U* test, depending on the distribution of the variable. Differences in the occurrence of AEs between each group were calculated using the Fisher's exact test. In the statistical analyses, *p*‐values lower than 0.03 were considered statistically significant after correction for multiple comparisons using the Benjamini and Hochberg method for false discovery rate.^16^ Cohen's H was used to calculate effect sizes, which describe the practical significance of the differences between the two populations. Effect sizes are classified as small (*d* = 0.2), medium (*d* = 0.5) and large (*d* = 0.8).^17^ Analyses were conducted with SPSS Statistics (version 28). Figures were created using BioRender.nl, Graphpad Prism (version 9) and Excel (version 2013).

## 3 RESULTS

### 3.1 Patient characteristics

**FIGURE 1:**
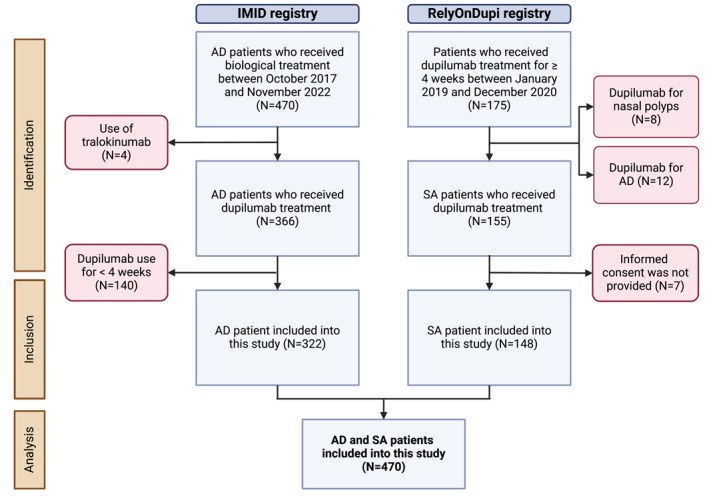
Flowchart of the study design. AD, atopic dermatitis; SA, severe asthma.

A total of 322 AD patients from the IMID registry and 148 SA patients from the RelyOnDupi registry were included in the analysis of this study, giving a total of 470 patients (Figure 1). The median follow‐up time was up to 24 months [24–24]. 50.6% were female with a median age of 43 [27.0–56.0] years and mean BMI of 26.8 (±6.1 kg/m^2^). SA patients had a significantly higher median age (52.5 years, *p* < 0.001), mean BMI (29.1 kg/m^2^, *p* < 0.001) and included more females (61.5%, *p* = 0.002) at baseline compared with AD patients. AD patients had a significantly higher prevalence of allergic conjunctivitis compared with SA patients (54.7%, *p* < 0.001) at baseline (Table 1). Furthermore, 27.3% AD patients had concomitant mild to moderate (GINA 1–4) asthma, whereas 38.5% SA patients had concomitant mild to severe AD. Fifty‐eight SA patients (39.2%) were using maintenance OCS at baseline: 43.1% less than 10 mg a day, 44.8% 10–20 mg a day and 5.2% more than 20 mg a day.

**TABLE 1:** Patient characteristics of AD and SA patients.

	Total (*N* = 470)	AD patients (*n* = 322)	SA patients (*n* = 148)	*p* Value
Median age [IQR], years	40.0 [27.0–56.0]	33.4 [24.7–50.0]	52.5 [38.3–63.8]	*p* < 0.001
Mean BMI (+/− SD), kg/m^2^	26.8 (+/−6.1)**	25.3 (+/−5.9)^a^	29.1 (+/−5.6)	*p* < 0.001
Female, *N* (%)	238 (50.6%)	147 (45.7%)	91 (61.5%)	*p* = 0.002
Asthma, *N* (%)	236 (50.2%)	88 (27.3%)	148 (100%)	*p* < 0.001
Atopic dermatitis, *N* (%)	379 (80.6%)	322 (100%)	57 (38.5%)	*p* < 0.001
Allergic rhinitis, *N* (%)	327 (69.6%)	231 (71.7%)	96 (64.9%)	*p* = 0.160
Allergic conjunctivitis, *N* (%)	221 (47.0%)	176 (54.7%)	45 (30.4%)	*p* < 0.001

Abbreviations: AD, atopic dermatitis; n, number; SA, severe asthma.

^a^ 88 missing values.

### 3.2 Adverse events

**FIGURE 2:**
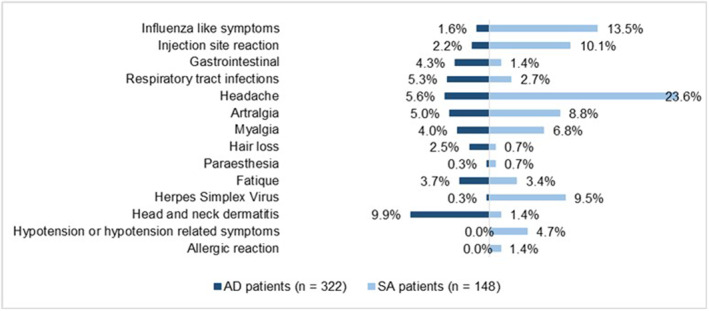
Adverse events of AD and SA patients during dupilumab treatment. AD, atopic dermatitis; SA, severe asthma.

In the total population (*n* = 470), 295 patients (62.8%) experienced at least one AE. The most frequently reported AEs were headaches (11.3%), head and neck dermatitis (7.2%), arthralgia (6.2%), influenza‐like symptoms (5.3%) and myalgia (4.9%). Significantly more AD patients (70.5%) developed one or more AEs compared with SA patients (45.9%) (*p* < 0.001), which was a medium effect between the two groups. Headaches (23.6%), influenza‐like symptoms (13.5%) and injection site reactions (10.1%) were significantly more frequently reported in SA patients compared with AD patients (*p* < 0.001). Fifteen SA patients (10.1%) developed injection site reactions; all patients used prefilled syringes. Seven (2.2%) AD patients developed injection site reactions, including four patients (57.1%) using prefilled syringes and three (42.9%) patients using auto‐injectors. In AD patients, head and neck dermatitis was more frequently reported compared with SA patients (9.9%) (*p* < 0.001) (Figure 2, Table S1). Furthermore, no significant differences in AEs were found between SA patients receiving 200 mg and those receiving 300 mg of dupilumab (*p* = 0.142). In SA patients, eosinophilia (eosinophils ≥500/μL) and hypereosinophilia (eosinophils ≥1500/μL) was observed in 13 patients (8.8%) and four patients (2.7%), respectively. Therapeutic intervention was unnecessary as the eosinophil levels decreased over time. Furthermore, a mean weight gain of 0.7 (±5.3 kg) was observed in SA patients (*n* = 120) between baseline and the end of the follow‐up (*p* = 0.077).

### 3.3 Ocular adverse events

Table 2 shows the reported ocular AEs in the total population. The total number of ocular AEs in this study was 506. In AD patients, 485 (95.8%) ocular AEs were observed, compared with 21 (4.2%) in SA patients. In the total population, one or more ocular AEs were observed in 217 patients (46.2%), including 200 AD patients (92.2%) and 17 SA patients (7.8%) (*p* < 0.001), which is a significant effect. These significant differences were also found even after adjusting for atopic SA.

**TABLE 2:** Ocular adverse events of AD and SA patients.

	Total *N* = 470	AD patients *n* = 322	SA patients *n* = 148	*p* Value	Effect size
Ocular adverse events, total	506	485	21		
Total patients with ≥1 ocular adverse event, *n* (%)	217 (46.2%)	200 (62.1%)	17 (11.5%)	*p* < 0.001	*h* = 1.12
Signs (*n* = 470), *n* (%)					
Irritation	58 (12.3%)	57 (17.7%)	1 (0.7%)	*p* < 0.001	*h* = 0.70
Redness	68 (14.5%)	68 (21.1%)	0 (0%)	*p* < 0.001	*h* = 0.95
Tearing	56 (11.9%)	56 (17.4%)	0 (0%)	*p* < 0.001	*h* = 0.86
Itching	101 (21.5%)	100 (31.1%)	1 (0.7%)	*p* < 0.001	*h* = 1.02
Photophobia	4 (0.9%)	4 (1.2%)	0 (0%)	*p* = 0.313	*h* = 0.20
Swelling	12 (2.6%)	12 (3.7%)	0 (0%)	*p* = 0.022	*h* = 0.30
Dryness	103 (21.9%)	100 (31.1%)	3 (2.0%)	*p* < 0.001	*h* = 1.10
Blurred vision	18 (3.8%)	15 (4.7%)	3 (2.0%)	*p* = 0.203	*h* = 0.34
DAOSD diagnosis, *n* (%)					
Conjunctivitis	66 (14.0%)	55 (17.1%)	11 (7.4%)	*p* = 0.004	*h* = 0.30
Blepharitis	19 (4.0%)	18 (5.6%)	1 (0.7%)	*p* = 0.010	*h* = 0.31
Keratitis	1 (0.2%)	0 (0%)	1 (0.7%)	*p* = 0.315	*H* = 0.17

Abbreviations: AD, atopic dermatitis; DAOSD, dupilumab‐associated ocular surface disease; *n*, number; SA, severe asthma.

AD patients reported more ophthalmic signs than SA patients, including irritation (17.7%) (*p* < 0.001), redness (21.1%) (*p* < 0.001), tearing (17.4%) (*p* < 0.001), itching (31.1%) (*p* < 0.001), photophobia (1.2%) (*p* = 0.313), swelling (3.7%) (*p* = 0.022), dryness (31.1%) (*p* < 0.001) and blurred vision (4.7%) (*p* = 0.203). AD patients demonstrated a significantly higher prevalence of DAOSD diagnosis compared to SA patients, including conjunctivitis (17.1%, *p* = 0.004) and blepharitis (5.6%, *p* = 0.010).

The overall incidence of ocular AEs in SA patients showed no significant difference between patients who were using maintenance OCS and those not using maintenance OCS (*p* = 0.797). In addition, there were no significant differences in ocular AEs between SA patients using high‐dose inhaled corticosteroids (ICS) and those using low‐ and medium‐dose ICS (*p* = 0.076).

### 3.4 Ocular adverse events of severe asthma patients with concurrent atopic dermatitis

**FIGURE 3:**
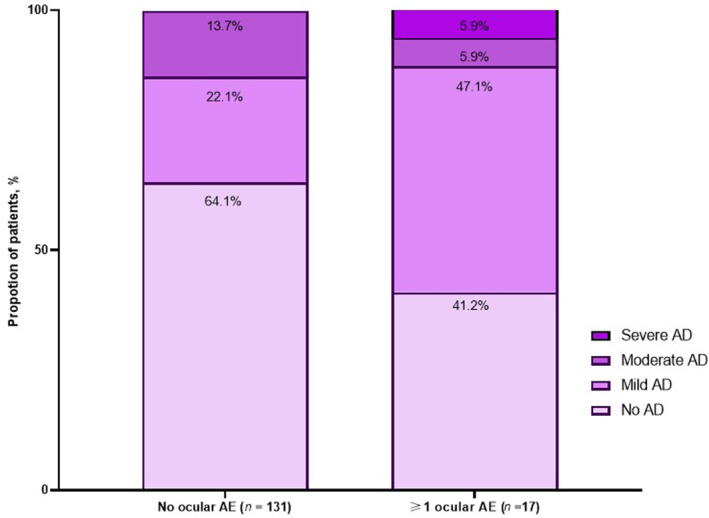
Proportion of ocular adverse events in SA patients based on concurrent AD. AD, atopic dermatitis; AE, adverse event; SA, severe asthma.

Concomitant AD was observed in 58.9% SA patients with ocular AEs, including 11.8% patients with moderate‐to‐severe AD. Interestingly, 88.5% SA patients had no ocular AEs, including 35.9% with concomitant AD. In this subgroup, 61.7% had mild AD, 38.3% had moderate AD and none of the patients had severe AD (Figure 3).

### 3.5 Onset of ocular adverse events in atopic dermatitis patients

In AD patients, the first ocular AE occurred after a median of eleven [4–24] weeks. Most patients (60.5%) experienced the first ocular AE in the first 12 weeks of treatment, while 15% experienced symptoms between 12 and 24 weeks. Additionally, 20.5% reported the first AEs after 24–52 weeks and 4% after more than 52 weeks of treatment (see also Figure S1).

### 3.6 Ophthalmic treatment

Most AD patients with ocular AEs received one or more ophthalmic treatments (88%), including lubricant eye drops (76.5%) and antihistamine eye drops (71.0%). Other therapies included tacrolimus ointments of the eyelids (24.5%), steroidal eye drops (11.5%), antibiotic eye ointments (2.5%), cyclosporine A eye drops (0.5%) and NSAID eye drops (0.5%). A high percentage of patients (38%), despite having lubricants and antihistamine eye drops with limited effectiveness, were referred to the ophthalmologist for further analysis and treatment. In SA patients with ocular AEs, 47% received one or more ophthalmic treatments, including lubricant eye drops (35.4%) and antihistamine eye drops (17.6%). 23.5% SA patients with ocular AEs were referred to the ophthalmologist (Table 3).

**TABLE 3:** Ophthalmic treatments of ocular adverse events.

	AD patients *n* = 200	SA patients *n* = 17
≥1 ophthalmic treatment, *n* (%)	178 (89.0%)	8 (47.0%)
• Lubricants	153 (76.5%)	6 (35.3%)
• Antihistaminic eye drops	142 (71.0%)	3 (17.6%)
• Steroidal eye drops	23 (11.5%)	0 (0%)
• Cyclosporine eye drops	1 (0.5%)	0 (0%)
• NSAID eye drops	1 (0.5%)	0 (0%)
• Antibiotic eye ointment (chloramphenicol)	5 (2.5%)	0 (0%)
• Tacrolimus ointment eyelids	49 (24.5%)	0 (0%)
• No action	71 (35.5%)	9 (52.9%)

Abbreviations: AD, atopic dermatitis; *n*, number; NSAID, non‐steroidal anti‐inflammatory drugs; SA, severe asth.

### 3.7 Dupilumab discontinuation

In the overall population (*n* = 470), dupilumab treatment was discontinued in 151 patients (32.1%). In AD patients (*n* = 322), 116 (36.0%) discontinued dupilumab treatment of which 40 (34.4%) were due to ocular AEs. Notably, of the 200 AD patients who experienced ocular AEs, 20% discontinued dupilumab treatment although 95% were receiving one or more ophthalmic treatments. Furthermore, 65% AD patients visited the ophthalmologist before discontinuing dupilumab due to ocular AEs. The most common diagnoses provided by the ophthalmologists before discontinuation of dupilumab treatment included keratoconjunctivitis sicca (*n* = 42.9%), allergic keratoconjunctivitis (27.0%) and blepharoconjunctivitis (*n* = 11.5%). After discontinuation of dupilumab due to ocular AEs, 35% switched to another biological (tralokinumab), 45% switched to Janus Kinase Inhibitors (abrocitinib, baricitinib, upadacitinib), 7.5% switched to classic systemic immunosuppressants (cyclosporine A, myfortic), 2.5% started ultraviolet‐B phototherapy and 10% continued with topical treatments only. Ocular AEs improved in 62.5% of patients after switching treatment. In contrast, most AD patients with ocular AEs (80%) continued dupilumab treatment, with 86.3% receiving one or more ophthalmic treatments and 31% being referred to an ophthalmologist, demonstrating a favorable treatment response.

In SA patients (*n* = 148), 35 (23.6%) discontinued dupilumab treatment. Two patients (5.7%) discontinued treatment due to an allergic reaction and three patients (8.6%) discontinued treatment due to ocular AEs, including one patient who received ophthalmic treatment. However, none of these patients who discontinued dupilumab due to ocular AEs were referred to an ophthalmologist for further evaluation. After discontinuation of dupilumab due to ocular AEs, one patient switched to mepolizumab, one patient switched to benralizumab, and one patient did not start any other biological treatment. Ocular AEs improved in all patients after switching treatment. In contrast, in SA patients with ocular AEs (*n* = 17) the majority continued dupilumab treatment (82.4%), with 64.3% receiving one or more ophthalmic treatments and 28.6% being referred to an ophthalmologist.

## 4 DISCUSSION

Using two large daily practice cohort studies to compare AEs in dupilumab‐treated AD and SA patients, we found a significantly higher incidence of ocular AEs in AD patients compared with SA patients. The pathogenesis of DAOSD is not fully understood. Several hypotheses have been posed, including a reduction in goblet cells, mucin deficiency and increased numbers of TH1 cells. This may be the result of a shift toward Th1 resulting from the inhibition of T2 signaling by dupilumab.^18^, ^19^ However, ocular AEs were found to be less common in SA patients, suggesting that additional mechanisms play a role.

AD patients receiving dupilumab treatment are known to have an increased risk of developing ocular AEs. Clinical trials showed ocular AE incidences in AD patients up to 22.1%.^10^ Ocular AEs were low in asthma clinical trials, which is in line with our study.^7^, ^8^ In SA patients, ocular AEs were more common with concurrent mild to severe AD, whereas SA patients without ocular AEs only had mild and moderate AD. This suggests that the severity of AD might be related to the development of ocular AEs. Both clinical trials and daily practice studies have shown associations between a higher baseline Eczema Area and Severity Index score and the development of ocular AEs in AD patients.^10^, ^12^ In our study, AD patients were initially treated with classic systemic immunosuppressants. If disease control is insufficient, new targeted therapies like dupilumab may be required.^20^ It is likely that these difficult‐to‐treat AD patients had a high severity score at the start of dupilumab treatment, which may explain the high incidence of ocular AEs in this study. Furthermore, AD is associated with a higher prevalence of ophthalmic comorbidities, including a previous history of conjunctivitis, which has been identified as a risk factor for the development of DAOSD.^10^ Therefore, AD patients may have a predisposition to develop DAOSD, or DAOSD may be an exacerbation of pre‐existing milder ophthalmic comorbidities. In this study, a significantly higher percentage of AD patients had a history of patient‐reported allergic conjunctivitis compared with SA patients, which may explain the higher incidence of ocular AEs in AD patients. However, 62.5% of AD patients and 100% of SA patients experienced an improvement in ocular AEs after discontinuing dupilumab treatment. This suggests that not only a previous history of conjunctivitis but also dupilumab treatment plays a role in the development of ocular AEs. Finally, 99.3% of SA patients were using ICS and 39.2% were on maintenance OCS, which may induce systemic effects and potentially suppress ocular inflammation in SA patients.^21^, ^22^ However, no significant difference in ocular AEs was observed between high‐dose ICS or maintenance OCS users and non‐users.

Remarkably, a subset of AD and SA patients with ocular AEs was not referred to an ophthalmologist, even when dupilumab was discontinued due to ocular AEs. We think that increased collaboration between pulmonologists, dermatologists, ophthalmologists, and potential allergologists is essential for better management of DAOSD. Improved collaboration between these specialties may lead to prolonged treatment with dupilumab in patients with good clinical response but experiencing ocular AEs.

SA patients experienced significantly more headaches, influenza‐like symptoms, and injection site reactions than AD patients. The high incidence of these AEs in SA patients may be related to the exclusive clinical administration of dupilumab injections during the initial 3 months, before at‐home self‐administration was permitted. These patients are closely monitored after dupilumab injections, which may lead to more frequent reporting of AEs. Moreover, in‐hospital administration of dupilumab may increase stress‐related headaches compared with home self‐administration, possibly due to the hospital environment. In addition, the increased incidence of influenza‐like symptoms and headaches in SA patients may be related to a higher prevalence of upper respiratory comorbidities in this group.^23^ In addition, SA patients developed a higher incidence of injection site reactions, which may be due to the use of prefilled syringes only, whereas AD patients who developed injection site reactions used prefilled syringes or auto‐injectors. However, previous research suggests that there is no association between the type of injector used and an injection site reaction.^24^ Finally, no clinically relevant weight gain was observed in SA patients in this study, in contrast to previous findings suggesting dupilumab‐induced weight gain, possibly through brown fat activation by the inhibition of IL‐4 and IL‐13.^25^ Further long‐term research is needed to investigate dupilumab‐induced weight gain.

In this study, paradoxical head‐and‐neck dermatitis related to dupilumab was frequently reported as an AE in AD patients, which is in line with previous studies.^12^, ^26^ The underlying pathogenesis of this phenomenon is not clear. Various hypotheses have been proposed, most of them related to a shift toward Th1/Th17‐driven responses caused by the inhibition of the T2 pathway by dupilumab.^27^, ^28^, ^29^ The higher incidence of head‐and‐neck dermatitis in AD patients suggests the absence of skin‐specific factors in SA patients. Furthermore, dermatologists may prioritize skin symptoms over pulmonologists, leading to more frequent reports of head‐and‐neck dermatitis in AD patients.

The current study provides important information about (ocular) AEs in AD and SA patients. However, several limitations should be considered. We compared data collected in two different hospitals, which may lead to a lack of uniformity in the registration of AEs. However, the effect sizes of most ocular AEs were moderate to high, which means that the two patient populations are comparable and the findings have practical significance. Comparing AD and SA patients is challenging due to the different dosing regimens. SA patients receive either 200 or 300 mg of dupilumab every 2 weeks, while AD patients receive 300 mg only. However, our current study does not show a significant difference in AEs between these doses, suggesting that different doses of dupilumab may not have an impact on the development of AEs. However, further research with larger patient cohorts is needed to confirm these findings. Furthermore, no comprehensive ophthalmic assessments by an ophthalmologist were performed before starting and during dupilumab treatment. As a result, the presence of pre‐existing ophthalmic pathology and the severity of ocular AEs were not determined in all patients. We found that 89% of the AD patients with ocular AEs received ophthalmic treatment(s) after the initiation of dupilumab treatment, which suggests that these AEs occurred after or worsened after the initiation of dupilumab treatment. In SA patients, only 47% received ophthalmic treatment(s), suggesting either a milder ocular disease or a lower likelihood of pulmonologists initiating ophthalmic treatments. Most AD and SA patients who experienced ocular AEs continued dupilumab treatment, indicating that the AEs were mild, the treatment was effective and/or there was spontaneous improvement.

### 4.1 Conclusion

In conclusion, dupilumab‐treated AD patients had a significantly higher incidence of ocular AEs compared with SA patients. Healthcare professionals should therefore be aware of the development of ocular AEs in patients with moderate to severe AD and in patients with SA and concomitant moderate to severe AD starting dupilumab treatment. Shared decision‐making with these patients is essential when considering initiating treatment with dupilumab, and multidisciplinary collaboration is necessary when ocular AEs occur. Furthermore, there is a need to better understand the underlying mechanisms and identify predictors of patients at higher risk of severe ocular AEs. These patients may benefit from early or preventive intervention for ocular AEs or may require an alternative therapeutic approach.

## AUTHOR CONTRIBUTIONS

**Anne R. Schlosser**: Conceptualization; Investigation; Methodology; Writing – original draft; Formal analysis; Visualization; Project administration; Data curation. **Lotte Bult**: Conceptualization; Investigation; Methodology; Writing – original draft; Formal analysis; Visualization; Project administration; Data curation. **John C. Thelen**: Writing – review & editing; Investigation; Data curation. **Alberta A. H. J. Thiadens**: Writing – review & editing. **Renske Schappin**: Formal analysis; Writing – review & editing. **Tamar E. C. Nijsten**: Writing – review & editing. **Johannes C. C. M. in 't Veen**: Supervision; Writing – review & editing. **Gerrit J. Braunstahl**: Supervision; Writing – review & editing. **DirkJan Hijnen**: Writing – review & editing; Supervision.

## CONFLICT OF INTEREST STATEMENT

AS: none; LB: Teva, unrestricted grant to faculty: Sanofi; JT: none; AT: none; RS: none; GJB: AstraZeneca, Teva, Sanofi, GSK, ALK, Novartis, Glaxo Smith Kline, Sanofi, Chiesi, AstraZeneca; HV: Unrestricted grants to faculty: Chiesi, Teva, Astra Zeneca. Speaker bureau: Sanofi, Chiesi, GSK, Astra Zeneca, Health Investment, Stichting RoLeX; TN: none; DJH: investigator for AbbVie, Almirall, LEO pharma, AstraZeneca, Galderma, LEO pharma, Novartis, Sanofi and consultancies for Abbvie, AstraZeneca, Janssen, LEO pharma, Lilly, Novartis, Pfizer, Sanofi.

## Supporting information

Supporting Information S1

## Data Availability

The data that support the findings of this study are available from the corresponding author upon reasonable request.
