# Correction to “Higher Prevalence of Dupilumab‐Induced Ocular Adverse Events in Atopic Dermatitis Compared to Asthma: A Daily Practice Analysis”

**DOI:** 10.1002/clt2.70154

**Published:** 2026-02-16

1

A. R. Schlösser, L. Bult, J. C. Thelen, et al., “Higher Prevalence of Dupilumab‐Induced Ocular Adverse Events in Atopic Dermatitis Compared to Asthma: A Daily Practice Analysis,” *Clinical and Translational Allergy* (2024): e12386, https://doi.org/10.1002/clt2.12386.

In the results of the Abstract section, the text ‘Additionally, 20% of AD and **17.6%** of SA patients discontinued dupilumab treatment…’ was incorrect. The correct value for SA patients should be **8.6%** instead of 17.6%. This is accurately reflected later in the main text. Fortunately, this typo does not affect any conclusions or other major aspects of the article, and the overall findings remain unchanged.

We apologize for this error.

