# Supplementary material for: Higher prevalence of dupilumab‐induced ocular adverse events in atopic dermatitis compared to asthma: A daily practice analysis
Source: Clin Transl Allergy. 2024 Aug 16;14(8):e12386. doi: 10.1002/clt2.12386 (PMC11329365; doi:10.1002/clt2.12386)
Supplement: Supplementary file 1 — Supporting Information S1 [file CLT2-14-e12386-s001.docx]

# **SUPPORTING INFORMATION**

Supplementary Table 1: Adverse events of AD and SA patients

|  | **Total (N = 470)** | **AD patients (n = 322)** | **SA patients (n = 148)** | **p value** | **Effect size** |
| --- | --- | --- | --- | --- | --- |
| **Total patients, n (%)** | 295 (62.8%) | 227 (70.5%) | 68 (45.9%) | p<0.001 | h=0.50 |
| **Influenza like symptoms, N (%)** | 25 (5.3%) | 5 (1.6%) | 20 (13.5%) | p<0.001 | h=0.50 |
| **Injection site reaction, N (%)** | 22 (4.7%) | 7 (2.2%) | 15 (10.1%) | p<0.001 | h=0.35 |
| **Gastrointestinal, N (%)** | 16 (3.4%) | 14 (4.3%) | 2 (1.4%) | p=0.108 | h=0.18 |
| **Respiratory tract infections, N (%)** | 21 (4.5%) | 17 (5.3%) | 4 (2.7%) | p=0.240 | h=0.13 |
| **Headache, N (%)** | 53 (11.3%) | 18 (5.6%) | 35 (23.6%) | p<0.001 | h=0.54 |
| **Artralgia, N (%)** | 29 (6.2%) | 16 (5.0%) | 13 (8.8%) | p=0.147 | h=0.15 |
| **Myalgia, N (%)** | 23 (4.9%) | 13 (4.0%) | 10 (6.8%) | p=0.249 | h=0.12 |
| **Hair loss, N (%)** | 9 (1.9%) | 8 (2.5%) | 1 (0.7%) | p=0.284 | h=0.15 |
| **Paraesthesia, N (%)** | 2 (0.4%) | 1 (0.3%) | 1 (0.7%) | p=0.531 | h=0.06 |
| **Fatigue, N (%)** | 17 (3.6%) | 12 (3.7%) | 5 (3.4%) | p=1.000 | h=0.02 |
| **Herpes Simplex Virus, N (%)** | 15 (3.2%) | 14 (4.3%) | 1 (0.7%) | p=0.045 | h=0.52 |
| **Head and neck dermatitis, N (%)** | 34 (7.2%) | 32 (9.9%) | 2 (1.4%) | p<0.001 | h=0.40 |
| **Hypotension or related symptoms** | 7 (1.5%) | 0 (0.0%) | 7 (4.7%) | p<0.001 | h=0.44 |
| **Allergic reaction** | 2 (0.4%) | 0 (0.0%) | 2 (1.4%) | p=0.099 | h=0.24 |

Abbreviations: AD, atopic dermatitis; n, number; SA, severe asthma

Supplementary Figure 1: Onset of first ocular adverse event in AD patients during dupilumab treatment


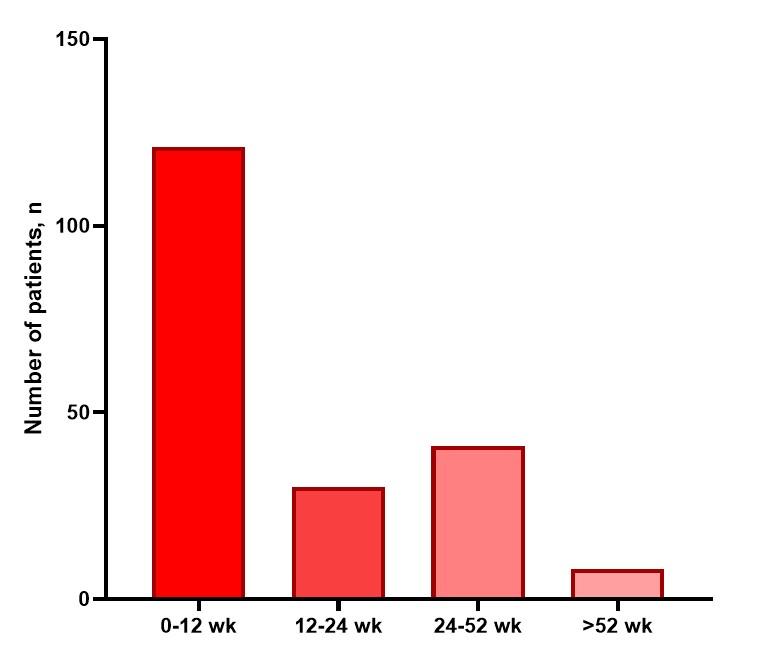


Abbreviations: AD, atopic dermatitis
